# Adverse Drug Reactions and Potentially Inappropriate Medication in Older Patients: Analysis of the Portuguese Pharmacovigilance Database

**DOI:** 10.3390/jcm11082229

**Published:** 2022-04-15

**Authors:** Daniel Gomes, Maria Teresa Herdeiro, Inês Ribeiro-Vaz, Pedro Lopes Ferreira, Fátima Roque

**Affiliations:** 1 Research Unit for Inland Development, Polytechnic of Guarda (UDI-IPG), 6300-559 Guarda, Portugal; 2 Centre for Health Studies and Research, University of Coimbra, 3004-512 Coimbra, Portugal; pedrof@fe.uc.pt; 3 Institute of Biomedicine, University of Aveiro (iBIMED-UA), 3810-193 Aveiro, Portugal; 4 Porto Pharmacovigilance Centre, Faculty of Medicine, University of Porto, 4200-319 Porto, Portugal; inesvaz@med.up.pt; 5 Center for Health Technology and Services Research (CINTESIS), Faculty of Medicine, University of Porto, 4200-319 Porto, Portugal; 6 Faculty of Economics, University of Coimbra (FEUC), 3004-512 Coimbra, Portugal; 7 Health Sciences Research Centre, University of Beira Interior (CICS-UBI), 6200-506 Covilhã, Portugal

**Keywords:** pharmacovigilance, adverse drug reactions, potentially inappropriate medication, older patients

## Abstract

Criteria have been developed to identify potentially inappropriate medications that can enhance adverse reactions, highly prevalent in older patient’s therapy. This study aimed to identify potentially inappropriate medications within the adverse drug reactions reported in the Portuguese pharmacovigilance system, characterizing the reports where inappropriate medications were identified. INFARMED, I.P. provided all adverse drug reactions reported from January to December 2019 in 65-year-old and older patients. Adverse drug reactions were characterized according to the System Organs Classes, seriousness, and medications with the Anatomical Therapeutical Classification. Potentially inappropriate medications were identified by applying the EU-(7)-PIM and the Beers criteria. A *p* value < 0.05 was considered statistically significant. From the 2337 reports considered for the analysis, PIMs were found in 12.8% of these, and 64.7% of all adverse reaction reports were classified as serious. Within the group of reports including at least one PIM, 71.4% were classified as serious, with hospitalization the most common criteria (35.1%). From the 3170 suspected medicines identified, 10.6% were classified as PIMs. Amiodarone was the most frequent PIM identified in the study (10.1%). Reports including at least one PIM were more associated with a higher number of ADRs (*p* = 0.025) reported in the same record, higher number of suspected medicines identified (*p* < 0.001), seriousness (*p* = 0.005), and hospitalization (*p* < 0.001). Potentially inappropriate medications are important enhancers of serious adverse drug reactions, increasing the likelihood of hospitalizations. This reinforces the importance of improving medication appropriateness in the older population.

## 1. Introduction

An aging population is a profound demographic transformation worldwide, particularly in Portugal, one of the most ageing countries in the world [1]. Physiological and cognitive changes inherent to the aging process can cause significant modifications in the pharmacokinetic and pharmacodynamic processes [2]. Thus, particularly if polymedicated, older adults become much more susceptible to drug interactions, and they experience an aggravated risk of possible adverse drug reactions (ADRs), more than any other age group [3,4]. ADRs not only cause a significant burden on healthcare services but also a strong economic impact on the healthcare system [5].

For every medicine, a balance between therapeutic efficacy and safety risks needs to be assessed. If the potential risk of a medicine exceeds the potential benefit, and for which there are safer alternatives, it is considered potentially inappropriate (PIMs) [6], which is highly prevalent in older patients medication therapy [3,7]. These prescriptions enhance adverse reactions, with a significant impact on hospital admissions, increasing healthcare expenditure [3]. These medicines are already identified from various tools developed, with the most used in published studies being the explicit Beers criteria [8] and the START/STOP criteria, which require additional patient data [9]. More recently, new explicit criteria were published, the EU-7-PIM list [10]. Thus, many of these adverse drug events or adverse drug reactions can be prevented in the prescription process [3]. Since this age group is more susceptible to being polymedicated, associated with chronic diseases and comorbidities, it is essential to continuously monitor safety and evaluate the benefit/risk of these medicines [11,12,13].

In Portugal, all serious or unexpected ADRs must be reported by healthcare professionals, as well as by the pharmaceutical industries and documented by regional units or by the National Authority on Medicines and Health Products (INFARMED, IP). Patients are also able to report ADRs. The Portuguese Pharmacovigilance System allows the safety monitoring of medicines through various methods. The spontaneous report (as a hypothesis-generating method) is the most used method by health professionals and patients, where an occurrence (adverse reaction) associated with a suspected treatment (at least one suspected medication) used by one person is reported [14,15,16].

This study aimed to characterize the ADR reports received by the Portuguese Pharmacovigilance System of INFARMED, I.P. in patients 65 and over from January to December 2019, and to identify suspected medicines classified as PIMs applying the EU (7) PIM list and Beers criteria, analyzing in depth the reports including PIMs in suspected medication.

## 2. Materials and Methods

### 2.1. Study Design and Data Source

A retrospective observational study was designed, and the data source was the Portal RAM, INFARMED, IP database [17]. The information contained is under the responsibility of the reporter and the professionals who processed it. All ADR reports received from January to December 2019 were requested, concerning individuals aged 65 and over exposed to at least one suspected medicine, with a total of 2919 reports received. Each report corresponded to one patient. However, the same patient could have more than one ADR report notified during 2019.

### 2.2. ADR Report Characterization

The terminology used to code ADRs was based on the Medical Dictionary for Regulatory Activities (MedDRA^®^) [18], used by INFARMED, I.P, where medical terms are coded according to the Systems Organ Classes (SOC) affected [19]. If there was more than one ADR belonging to the same SOC in the same report, that SOC was counted only once. Regarding seriousness, ADR reports were characterized based on the definition of Good Pharmacovigilance Practices, Module VI [20], where a serious ADR is any reaction that causes death, is life-threatening, requires hospitalization or prolongation of existing hospitalization, results in persistent or significant disability or incapacity, or is a congenital anomaly/birth defect. The suspected medicines involved were characterized by therapeutic group according to the WHO Anatomical Therapeutic Chemical (ATC) classification system [21]. As the report may have more than one suspected medicine, the total number of ATCs considered may be higher than the number of reports analyzed.

### 2.3. PIM Identification

To identify PIMs, the operationalization for the Portuguese reality of the EU (7) PIM list by DA. Rodrigues et al. [22] was applied, as well as the most updated Beers criteria from 2019 [23]. It was not possible to apply the START/STOP criteria in this study as clinical information required was not available in any report.

Both lists were applied whenever possible according to the information provided by each ADR report. In some reports, it was possible to identify dosage and treatment duration, allowing the application of PIM criteria related to dosage and treatment duration. However, some reports did not contain this information. Whenever clinical information was needed to apply the criteria, such as renal function-related PIM, these could not be applied, as that information was not available in the database.

Criteria was applied to the available information in the database. Whenever the information on the report was not enough, suspected medicines could not be identified as PIM in that report. Information about the application of the criteria with the EU (7) PIM list and Beers 2019 is detailed in Supplementary Material (Tables S1 and S2).

### 2.4. Statistical Analysis

Quantitative variables were described by mean, median and standard deviation (SD). As distribution was not normal (Kolmogorov–Smirnov test: *p* < 0.001), the Chi-square test was used for comparison of two qualitative variables and Mann–Whitney was used for comparison of quantitative and qualitative variables. Data analysis was executed using the Statistical Package for Social Sciences (SPSS 25, IBM Corp., New York, NY, USA) and all p values ≤ 0.05 were considered statistically significant.

## 3. Results

### 3.1. ADR Report Characterization

After data cleaning for duplicate, rejected and nulled reports, a total of 2337 ADR reports were considered for the analysis. The study population had a mean age of 74.6 ± 6.8 years. Age ranged between 65 and 97 years and females represented 54.3% (*n* = 1189) of the reports, and 48.7% (*n* = 1137) of the reports were notified by physicians. In addition to physicians, nurses (4.9%/*n* = 114), pharmacists (17.1%/*n* = 399), and other healthcare professionals (21.1%/*n* = 493) were involved in reporting these ADRs. Users and non-health professionals participated in 14.4% (*n* = 337) of the reports.

Among the reports received, 64.7% (*n* = 1512) were classified as serious. Seriousness for being clinically important (*n* = 781) was the most common identified criteria, followed by hospitalization (*n* = 600), life threatening (*n* = 159), death (*n* = 114) and disability (*n* = 85).

In total, 6617 ADRs were identified (each report could have more than one ADR from the same SOC), meaning that each report had a mean of 2.83 ± 2.89 (min.1; max 36) ADRs. Table 1 shows the four most common SOCs reported, with “General Disorders and administration site conditions” the most frequent, identified in 28.7% (*n* = 671) of the reports, followed by “Skin and subcutaneous tissue disorders”, in 21.9% (*n* = 512), “Gastrointestinal disorders”, in 20.3% (*n* = 475), and “Nervous System disorders”, in 16.0% (*n* = 375).

Within the total reports, 3170 (min.1; max. 14) suspected medicines were identified, representing an average of 1.36 ± 1.07 drugs identified per report. As shown in Table 2, the ATC subgroups most often identified in the reports received during 2019 in older patients were group A10B—Oral Antidiabetics (*n* = 160/5.0%), B01A—antithrombotic agents (*n* = 193/6.1%) and L01X—Other antineoplastic agents (*n* = 275/8.7%).

### 3.2. PIM-Related ADR Reports

After applying the Beers and EU (7) PIM criteria, within the 2337 reports included in the study, PIM were found in 299 reports, representing 12.8% of the total reports. From the total 3170 suspected medicines identified, 337 (10.6%) were classified as PIM; 60.5% (*n* = 204) of PIM were recognized only by EU (7) PIM List, whereas 29 (8.6%) were recognized only by Beers. It was possible to identify 104 suspected medicines (30.9%) by both criteria instead of just one of the criteria. In Table S3 of the Supplementary Materials, it is possible to identify the ATC code of the PIM identity, the frequency, and what criteria identified the PIM (or if both).

Table 3 shows that Nervous System disorders were reported in 49.1% (*n* = 147) of the ADR reports including PIM, followed by “Skin and subcutaneous tissue disorders”, in 37.8% (*n* = 113), and “General Disorders and administration site conditions” in 32.1% (*n* = 92). Table 4 lists the pharmacological subgroups that were classified as PIM, with CO1B—Antiarrhythmics, class I and III (28.3%/*n* = 95), B01A—Antithrombotic agents (18.8%/*n* = 63), M01A—Anti-inflammatory and antirheumatic products, non-steroids (14.3%/*n* = 48), N05A—Antipsychotics (8.6%/*n* = 29), N05B—Anxiolytics (7.4%/*n* = 25) and N06A—Antidepressants (5.4%/*n* = 18). As available in the Supplementary Material (Table S3), Amiodarone (C01BD01) was the most frequent (*n* = 34/10.1%), followed by dabigatran etexilat (B01AE07) with 7.1% (*n* = 24) and Rivaroxaban (B01AF01) with 6.2% (*n* = 21).

Older patients reporting PIM in suspected medicines showed a mean age of 73.0 ± 6.6 years (min. 65; max 95), with 56.5% (*n* = 169) female (Table 5). In these reports, 45.2% (*n* = 135) were reported by physicians and 14.4% (*n* = 43) by pharmacists. Users and non-health professionals represented 26.1% (*n* = 76) of the reports received (Table 6). In total, 71.4% (*n* = 215) of these reports were classified as serious, with hospitalization the most common criteria with a percentage of 35.1% (*n* = 105), followed by being clinically important with 32.1% (*n* = 96), risk of life with 7.7% (*n* = 23), disability (4.3%/*n* = 13), and finally, death (2.7%/*n* = 8). It was possible to identify 1009 ADRs, with a mean of 3.37 ± 3.59 ADRs per report (min.1; max 20). 

Comparison analysis was made between the reports including PIM in suspected medicines (*n* = 299) and the ones who did not include PIM in the suspected medicines causing ADRs. Table 5 shows significant differences between groups in the distribution of the number of ADRs (*p* = 0.025) and in the number of suspected medicines identified in the reports (*p* < 0.001). Both variables were higher in the ADRs reporting PIM. On the other hand, no differences were found regarding sex and age of the patients.

In Table 6, variables were compared to find associations between these variables and reports where PIM were identified. Significantly stronger associations were found with ADRs reporting PIM in seriousness of the ADR (*p* = 0.005), as well as in the Hospitalization subclassification (*p* < 0.001). Furthermore, associations were also found in the reporters of the ADR, namely with nurses (*p* = 0.014) and patients or non-health professionals (*p* = 0.014).

Moreover, a stronger association was found with ADR reports containing PIM and the SOC Nervous System disorders (*p* < 0.001), Respiratory, thoracic, and mediastinal disorders (*p* = 0.002), Psychiatric disorders (*p* < 0.001) and Vascular disorders (*p* < 0.001), as presented in Table 7.

## 4. Discussion

Studying older patient’s spontaneous ADR reports is still uncommon but has been growing in the past few years [24,25]. This study intended to address this subject with data from the Portuguese pharmacovigilance system, not only describing the ADR reports involving older patients, but to identify potential inappropriate medication involved in these reports, helping to close the gap persisting in the scientific literature and updating the data. Until the time of development of the study, no article was found studying the Portuguese pharmacovigilance database to assess ADR in reports with PIM in older adults; however, a similar study had been performed in France [24]. The results obtained can bring new insights, providing a snapshot of the problem in Portugal and solutions to overcome it.

The majority of PIM were identified only with the EU (7) PIM list (60.5%), as some medications (30.9%) were listed in both criteria. Since the EU (7) PIM list used was an operationalization for the Portuguese reality for a criteria already based in European approved medicines, it is understandable that this list was able to identify more PIM [23]. Furthermore, the EU (7) PIM list requires less information about the patient’s clinical status, based only on the prescribed medication and also the dosage and treatment duration for some medicines. Moreover, it was impossible to apply significant part of the Beers criteria, as pathology and laboratory results of the patients were needed (see Supplementary Materials) [22,23]. Nevertheless, a study in Portugal concluded that there is a low overlap and agreement between these two tools and considering the number of PIM identified in the ADR reports, this only highlights the need to develop clinical decision support systems for PIM detection in a highly exposed older population, such as the Portuguese one [26].

In 2019, the Portuguese pharmacovigilance database received 2337 valid ADR reports related to older patients, an increasing trend that was already observed in another study in Portugal [25]. This can be explained from a demographic perspective, as the number of older adults has been increasing in Portugal [27]. However, the constant efforts of raising awareness for reporting ADRs also explain part of this increase [28,29].

Overall, most of the reports were in older women, reported by physicians, and were mostly classified as serious. “General disorders and administration site conditions” was the most identified SOC within the reports whereas within the L01X group, “Other neoplastic agents” was the most frequent group identified. Of the suspected medications, 10.6% were classified as PIM, and those were identified in 12.8% of the reports. Seriousness of the ADR was more associated with reports including PIM.

The majority of the reports were in older women patients, as seen in the literature [30,31]. Although a study in the center region of Portugal with older patients showed that female older patients are more adherent to medication than men, avoiding ADRs [32], which female patients tend to report more [33]. Furthermore, there are pharmacokinetics and pharmacodynamics differences between females and males, which may explain the increased risk of ADRs in women [33,34]. This reinforces the need for particular attention to these older patients in the medication appropriateness assessment. On the other hand, age seems to have no influence.

As expected, physicians play an important role in reporting ADRs to pharmacovigilance systems [35,36]. However, when we compare the reporters with the subgroup of reports including PIM as suspected medicine, a significant increase is observed in reports made by patients or other health professionals (*p* = 0.014), which could indicate that targeting healthcare professionals (namely GPs and pharmacists) to raise awareness in their older patients and caretakers could improve prescription reporting, particularly when PIMs are involved.

Almost two-thirds of the overall reports were considered serious. The tendency of healthcare professionals or others to report more serious ADRs that are easier to identify explains this high percentage [37]. In the group of reports including PIM, the percentage is even higher (71.9%), reinforcing the risk of morbidity and hospitalization when using PIMs [38]. These PIMs are prevalent in older patients’ medication consumption in the country, and general studies have shown an association with ADRs and hospital admissions [39,40,41,42]. Hospitalizations can be associated with a significant increase of suspect medicines identified in the reports including PIMs, potentiating inadequate prescriptions [43,44,45,46]. Adding to this, it was observed that the number of suspected medicines significantly increases when PIMs are included in the list (*p* < 0.001). Particularly in Portugal, a study in the centre region of the country using the EU (7) PIM list showed 83.7% of older patients taking at least one medicine were included in the final potentially inappropriate medicines list or belonging to one of the groups included in it [22]. A cross sectional study identified PIMs in 68.6% of the older adults in the sample and 46.1% of the sample had two or more [47]. All these data reinforce the importance of implementing medication review procedures to improve the quality of the prescription with educational programs, not only for the healthcare professional but also for patients [48,49,50]. As 12.8% of the ADR reports received by the pharmacovigilance database were PIM related, this effort in improving prescription quality can positively impact these outcomes [51].

The most frequently reported ADRs according to MedDRA SOC were general disorders and administration site conditions, skin and subcutaneous tissue reactions and gastrointestinal disorders, as shown in other studies [25,52]. These types of ADR are also frequent in the ADR group including PIM. However, ADRs related to Nervous System Disorders and Respiratory thoracic and mediastinal disorders were significantly more frequent (*p* < 0.001; *p* = 0.002) than the reports without PIM, as well as vascular (*p* < 0.001) and psychiatric disorders (*p* < 0.001). The most frequent ATCs identified in PIM are from the C—Cardiovascular group, as for example Amiodarone (10.1%/*n* = 34) (C01B—Antiarrhythmics, class I and III), M—Musculoskeletal group, such as Diclofenac (5.6%/*n* = 19) and N—Nervous System group, such as Haloperidol (3.6%/*n* = 12), which enhances those ADRs and explains these associations. A study in the Pharmacovigilance database in a region of France is aligned with our results, enhancing cardiovascular, nervous system medication and cardiovascular as most common PIMs identified [24]. These findings are particularly interesting when studies regarding the consumption of medicines in older patients also show most of these classes as the most frequently consumed [32]. Furthermore, PPIs, NSAIDs and benzodiazepines are among the most common PIMs in the older adult population in primary health care in Portugal [47].

### Limitations

Although it fills a gap in the literature, this study had some limitations. Based on the information of spontaneous ADR reports, the most important source of information for pharmacovigilance studies, even with all the efforts made by the pharmacovigilance units in Portugal to enhance reporting (with success), underreporting is still the biggest obstacle in these studies. Furthermore, there is a bias towards serious ADRs, since these reports are more likely to be reported than non-serious. This would aggravate the seriousness of PIM-related ADRs in a universe of ADR PIMs that were not reported in 2019 [17,37].

Another important limitation of this study comes from the lack of information from the ADR reports. In both lists for most of the criteria, clinical information of the patient or dosage and treatment duration is required. However, most reports did not mention the treatment period and dosage, hampering the applicability of the criteria in medication within the, for example, benzodiazepines, Proton Pump Inhibitors (PPI) or insulins as potentially inappropriate [23]. As this information was not available in the majority of cases, most medicines from some classes were not identified as PIM, and thus, those reports were also not classified as ADR reporting PIM. Particularly in the PPIs, recent studies have shown that they are overused in older patients and that 25% to 70% of prescriptions have no indication for use [44]. This means that the ADR reporting PIM group would have more reports if the medication profile information was always available. As shown in Supplementary Material (Table S4), it was not possible to apply the criteria in any of the insulins identified (*n* = 94) due to lack of information, as well as 85% (*n* = 28 in 33) of the PPIs. Adding to these, Ibuprofen (*n* = 15), Naproxen (*n* = 9) and Risperidone (*n* = 10) were identified as suspected medications, but it was not possible to apply the criteria in any of them.

It is also important to consider that the number for reports without PIM (*n* = 2038) is almost 10 times the number of reports including PIM (*n* = 299), and so statistical significance interpretation must be done cautiously.

With the data available, we were not able to confirm that a PIM was directly responsible for one or more ADR reported, but that the identified suspected medication in one report caused the mentioned ADR. However, it is possible to confirm with the data provided that reducing PIM would have a direct impact on reducing ADR in older patients, aligning with the available literature in other countries [24,53].

Finally, the data source was chosen to show findings on ADR and PIM in the Portuguese older adults’ population, not to assess age stratified differences among different populations, and so the comparison between older adults and younger patients is not available. Regardless, important data were obtained from the statistical treatment, even considering the gaps identified previously.

## 5. Conclusions

An important part of the ADR reports received by the Portuguese pharmacovigilance system included PIMs. These PIMs are enhancers of ADRs. This study highlighted the significant impact of PIMs on the seriousness of these ADRs and enhancing the likelihood of hospitalizations. Avoiding the consumption of these medicines already identified in different lists can help reduce medical costs associated with hospitalization or ADR treatment as well as improving the quality of life for older adults. The healthcare authorities need to actively promote the development and use of clinical decision support systems integrating the PIM criteria available to avoid these inappropriate prescriptions and, therefore, severe adverse reactions.

## Figures and Tables

**Table 1:** Descriptive statistics of the ADR reported according with SOC of MedDRA terminology.

ADR ^1^ According to MedDRA ^2^ SOC ^3^ Terminology (>15%)	N Reports Identified	%
General Disorders and administration site conditions	671	28.7%
Skin and subcutaneous tissue disorders	512	21.9%
Gastrointestinal disorders	475	20.3%
Nervous system disorders	375	16.0%

^1^ Adverse Drug Reaction; ^2^ Medical Dictionary for Regulatory Activities; ^3^ Systems Organ Classes.

**Table 2:** Descriptive statistics of the ATC groups reported as suspected medicines.

Pharmacological ATC ^1^ Subgroup (>3%)	N ATC ^1^ Identified	%
A10A—Insulin and analogues	94	3.0%
A10B—Blood glucose lowering drugs, excl. insulins	160	5.0%
B01A—Antithrombotic agents	193	6.1%
C10A—Lipid Modifying agents	95	3.0%
L01X—Other antineoplastic agents	275	8.7%
L04A—Immunosuppressants	117	3.7%
M01A—Anti-inflammatory and antirheumatic products, non-steroids	84	2.7%
N06A—Antidepressants	91	3.0%

^1^ Anatomical Therapeutic Classification.

**Table 3:** Descriptive statistics of the ADR according to the SOC of MedDRA terminology reported with PIM in suspected medicines.

ADR ^1^ According to MedDRA ^2^ SOC ^3^ Terminology (>15%)	N Reports Identified	%
General Disorders and administration site conditions	96	32.1%
Skin and subcutaneous tissue disorders	113	37.8%
Gastrointestinal disorders	92	30.8%
Nervous system disorders	147	49.1%
Respiratory disorders	78	26.1%

^1^ Adverse Drug Reaction; ^2^ Medical Dictionary for Regulatory Activities; ^3^ Systems Organ Classes.

**Table 4:** ATC classification of the PIM found in the ADR reports.

Pharmacological ATC ^1^ Subgroup (>3%)	N of Medicines Identified	%
A10B—Blood glucose lowering drugs, excl. insulins	11	3.3%
B01A—Antithrombotic agents	63	18.8%
C01B—Antiarrhythmics, class I and III	95	28.3%
M01A—Anti-inflammatory and antirheumatic products, non-steroids	48	14.3%
N03A—Antiepileptics	16	4.8%
N05A—Antipsychotics	29	8.6%
N05B—Anxiolytics	25	7.4%
N06A—Antidepressants	18	5.4%

^1^ Anatomical Therapeutic Classification.

**Table 5:** Differences between reports including PIM and without PIM, classified according with age, suspected medicines, ADR number reported and sex.

	Reports Including PIM ^1^ (*n* = 299)	Reports without PIM ^1^ (*n* = 2038)	*p* Value
Age	Mean: 75.3 ± 7.52; Median: 74	Mean: 74.46 ± 6.69; Median: 73	0.169
N of ADR ^2^	Mean: 3.37 ± 3.58; Median: 1	Mean: 2.75 ± 2.76; Median: 1	0.025
N of suspected medicines	Mean: 1.99 ± 2.16; Median: 2	Mean: 1.26 ± 0.752; Median: 2	<0.001
Sex	Female: 169 (56.5%)	Female: 1020 (50.0%)	0.10

^1^ Potentially Inappropriate Medication; ^2^ Adverse Drug Reaction.

**Table 6:** Differences between reports including PIM and without PIM, regarding seriousness, respective sub-classifications, and notifiers.

	Reports Including PIM ^1^ (*n* = 299)	Reports without PIM ^1^ (*n* = 2038)	*p* Value
Serious ADR ^2^ report	215 (71.9%)	1297 (63.6%)	0.005
Life risk	23 (7.7%)	136 (6.7%)	0.513
Hospitalization	105 (35.1%)	495 (24.3%)	<0.001
Death	8 (2.7%)	106 (5.2%)	0.058
Clinically relevant	96 (32.1%)	685 (33.6%)	0.607
Incapacity	13 (4.3%)	72 (3.5%)	0.482
Reported by physician	135 (45.2%)	1002 (49.2%)	0.195
Reported by pharmacist	43 (14.4%)	356 (17.5%)	0.185
Reported by nurse	6 (2.0%)	108 (5.3%)	0.014
Reported by patient or non-health professional	78 (26.1%)	415 (20.4%)	0.014

^1^ Potentially Inappropriate Medication; ^2^ Adverse Drug Reaction.

**Table 7:** Differences between the number of reports including PIM and without PIM, regarding most frequent SOC identified.

	Reports Including PIM ^1^ (*n* = 299)	Reports without PIM ^1^ (*n* = 2038)	*p* Value
Nervous System Disorders	82 (27.4%)	293 (14.4%)	<0.001
General disorders and administration site conditions	72 (24.1%)	599 (29.4%)	0.411
Skin and subcutaneous tissue disorders	67 (22.4%)	445 (21.8%)	0.823
Respiratory, thoracic, and mediastinal disorders	52 (17.4%)	230 (11.3%)	0.002
Gastrointestinal disorders	58 (19.4%)	417 (20.5%)	0.670
Psychiatric disorders	32 (10.7%)	83 (4.1%)	<0.001
Vascular disorders	41 (13.7%)	159 (7.8%)	<0.001

^1^ Potentially Inappropriate Medication.

## Data Availability

Restrictions apply to the availability of the data. Data was obtained from INFARMED, I.P. and the authors are not entitled to share it.

## Supplementary Materials

The following supporting information can be downloaded at: https://www.mdpi.com/article/10.3390/jcm11082229/s1, Table S1: Application of the American Geriatrics Society 2019 Updated AGS Beers Criteria^®^ for Potentially Inappropriate Medication Use in Older Adults in the study; Table S2: Application of the EU (7) -PIM list for the portuguese reality; Table S3: Total Number of PIM Identified In The ADR Reports Received from patients 65 and more years old during 2019; Table S4: Groups/Medicines included in the PIM lists that did not fulfil the criteria due to lack of information to apply it.
