# Supplementary material for: Adverse Drug Reactions and Potentially Inappropriate Medication in Older Patients: Analysis of the Portuguese Pharmacovigilance Database
Source: J Clin Med. 2022 Apr 15;11(8):2229. doi: 10.3390/jcm11082229 (PMC9029593; doi:10.3390/jcm11082229)
Supplement: Supplementary file 1 [file jcm-11-02229-s001.zip › jcm-1650228-supplementary.pdf]

Supplementary Material

Table S1. Application of the American Geriatrics Society 2019 Updated AGS Beers Criteria® for Potentially Inappropriate Medication Use in Older Adults in the study.

	**Group/Medicines**	**Application of criteria**
**Drugs To Be Used With Caution**	Aspirin for primary prevention of cardiovascular disease and colorectal cancer	Not applied, as clinical information was needed
	Dabigatran, Rivaroxaban	Not applied, as clinical information was needed
	Prasugrel	Not applied, as clinical information was needed
	Antipsychotics, Carbamazepine, Diuretics, Mirtazapine Oxcarbazepine, SNRIs, SSRIs, TCAs,Tramadol	Not applied, as use with caution recommendation would need clinical data that was not available
	Dextromethorphan/ quinidine	Not applied, as clinical information was needed
	Trimethoprimsulfamethoxazole	Not applied, as clinical information was needed
**Drug-Drug Interactions**	RAS inibithor – RAS inibithor	Not applied, as clinical information was needed
	Opioids - Benzodiazepines	Applied
	Opioids - Gabapentin, pregabalin	Not applied, as clinical information was needed
	Anticholinergic - Anticholinergic	Applied
	Any combination of three or more of Antidepressants (TCAs, SSRIs, and SNRIs), Antipsychotics, Antiepileptics, Benzodiazepines and nonbenzodiazepine, benzodiazepine receptor agonist hypnotics (ie, "Z-drugs"), Opioids	Applied
	Corticosteroids, oral or parenteral - NSAIDs	Applied
	Lithium - ACEIs	Applied
	Lithium - Loop diuretics	Applied
	Peripheral α-1 blockers - Loop diuretics	Not applied, as clinical information was needed
	Phenytoin - Trimethoprim-sulfamethoxazole	Applied
	Theophylline - Cimetidine	Applied
	Theophylline - Ciprofloxacin	Applied
	Warfarin - Amiodarone	Not applied, as for exception clinical data was needed
	Warfarin - Ciprofloxacin	Not applied, as for exception clinical data was needed
	Warfarin - Macrolides (excluding azithromycin)	Not applied, as for exception clinical data was needed
	Warfarin - Trimethoprim-sulfamethoxazole	Not applied, as for exception clinical data was needed
	Warfarin - NSAIDs	Not applied, as for exception clinical data was needed
**Drug-disease interaction**	Not applied as clinical information was needed	

**PIM Based on Kidney Function**	Not applied as clinical information was needed	
**Drugs With Strong Anticholinergic Properties**	Applied	
**Pottentially Innapropriate medication**	Anticholinergics	Applied
	Antiparkinsonian agents	Not applied, as clinical information was needed
	Antispasmodics	Applied
	Antithrombotics	Applied when type of administration available
	Anti-infective	Applied
	Cardiovascular	Applied expect when clinical was need: exclusion of Digoxin, Dronedarone, Amiodarone
	Antidepressants, alone or in combination	Applied
	Antipsychotics, first (conventional) and second (atypical) generation	Not applied, as clinical was needed
	Barbiturates	Applied
	Benzodiazepines	Applied
	Meprobamate	Applied
	Nonbenzodiazepine, benzodiazepine receptor agonist hypnotics (ie, “Z-drugs”)	Applied
	Ergoloid mesylates<br>(dehydrogenated ergot alkaloids)<br>Isoxsuprine	Applied
	Endocrine	Applied, expept when clinical information was needed: exclusion of Androgens, Estrogens, Growth Hormone, Insulin sliding scale,
	Gastrointestinal	Metoclopramide: not applied, as clinical was needed<br>Mineral oil: applied<br>Proton-pump inhibitors: applied, whenever as possible to confirm >8 weeks treatment
	Pain medications	Applied
	Genitourinary	Not applied, as clinical was needed

Table S2. Application of the EU (7) -PIM list for the portuguese reality.

Medicines	Application of Criteria
Magnesium Hydroxide	Applied
Aluminum compounds - Combinations and complexes of aluminum, calcium and magnesium compounds	Applied when dosage was available
Cimetidine	Applied
Ranitidine	Applied
Famotidine	Applied
Proton Bomb Inhibitors	Applied when information about treatment duration was available
Mebeverine	Applied
Trimebutine	Applied
Otilonium Bromide	Applied
Pinaverium	Applied
clidinium and psycholeptic	Applied
Metoclopramide	Applied
Domperidone (> 30 mg/d)	Applied when information about treatment duration was available
Liquid Parafine	Applied
Bisacodyl (> 3days)	Applied when information about treatment duration was available
Senna glycosided	Applied
Sodium picosulfate	Applied
Prucalopride	Applied
Loperamide (> 2 days)	Applied when information about treatment duration was available
Racecadotril	Applied when information about treatment duration was available
Insuline – only in "*sliding* scale"	Not applied as data not available
Glibenclamid	Applied
Glipizide	Applied when information about treatment duration was available
Glimepiride	Applied when information about treatment duration was available
Acarbose	Applied
Pioglitazone	Applied
Sitagliptin	Applied
Vildagliptin	Applied
Acenocumarol	Applied
Ticlopidine	Applied
Dipyridamole	Applied
Prasugrel	Applied
dabigatran etexilate	Applied
Rivaroxaban	Applied when information about treatment duration was available
Apixaban	Applied
Iron bivalent, oral preparations (> 325 mg/d)	Applied
Digoxin	Applied
Metildigoxin	Applied
Propafenone	Applied
Flecainide	Applied
Amiodarone	Applied
Dronedarone	Applied
Trimetazidine	Applied
Ivabradine	Applied
Clonidine	Applied
Guanfacine	Applied

Moxonidin	Applied
Rilmenidin	Applied
Doxazosin	Applied
Urapidil	Applied
Spironolactone (> 25 mg/d)	Applied
Pentoxifylline	Applied
Nicergoline	Applied
Vinburnine	Applied
Naftidrofurilo	Applied
Hidrosmina	Applied
Hesperidin + Ruscus aculeatus + ascorbic acid; Rutoside + ascorbic Acid	Applied
Troxerutin + Heparinoid	Applied
Propranolol	Applied
Sotalol	Applied
Labetalol	Applied when information about treatment duration and dosage was available
Nifedipine	Applied
Nifedipine (sustained release)	Applied
Verapamil	Applied
Diltiazem	Applied
Estrogens (oral)	Applied
Flavoxate	Applied
Oxybutynin	Applied
Solifenacin	Applied
Trospium chloride	Applied
Darifenacin	Applied
Fesoterodin	Applied
Terazosin	Applied
Ofloxacin	Applied
Prulifloxacin	Applied
Nitrofurantoin (> 1 week)	Applied when information about treatment duration and dosage was available
Indomethacin	Applied
Diclofenac	Applied
Etodolac	Applied
Acemetacin	Applied
Ketorolac	Applied
Aceclofenac	Applied
Piroxicam	Applied
Lornoxicam	Applied
Meloxicam	Applied
Ibuprofen (> 3 x 400 mg/d or > 1 week)	Applied when information about treatment duration and dosage was available
Naproxen (>2 x 250 mg/d or > 1 week)	Applied when information about treatment duration and dosage was available
Ketoprofen	Applied
Flurbiprofen	Applied
Dexcetoprofen	Applied
Acid mefenamic	Applied
Celecoxib	Applied
Etoricoxib	Applied when information about treatment duration and dosage was available
Nabumetone	Applied
Nimesulide	Applied
Baclofen	Applied when information about treatment duration and dosage was available

Tizanidine	Applied
Cyclobenzaprine	Applied when information about treatment duration and dosage was available
Colicine	Applied
Strontium ranelate	Applied
Pethidine	Applied when information about treatment duration and dosage was available
Tramadol	Applied when information about treatment duration and dosage was available
Tramadol (sustained release)	Applied when information about treatment duration and dosage was available
Acetylsalicylic Acid (> 325 mg)	Applied when information about dosage was available
Triptans	Applied when information about treatment duration and dosage was available
Phenobarbital	Applied when information about treatment duration and dosage was available
Phenytoin	Applied
Clonazepam	Applied when information about dosage was available
Carbamazepine	Applied
Topiramate	Applied
Trihexyphenidyl	Applied
Biperiden	Applied
Amantadine	Applied when information about dosage was available
Bromocriptine	Applied
dihydroergocryptine mesylate	Applied
Ropinirole	Applied when information about dosage was available
Pramipexol	Applied when information about treatment duration and dosage was available
Piribedil	Applied
Rotigotin	Applied when information about dosage was available
Selegiline	Applied when information about dosage was available
Chlorpromazine	Applied when information about dosage was available
Levomepromazine	Applied when information about dosage was available
Ciamemazin	Applied
Haloperidol (> 2 mg unic dose; >5 mg/d)	Applied when information about dosage and administration route was available
Droperidol	Not applied as clinical information was needed
Ziprasidone	Applied when information about dosage was available
Flupentixol	Applied
Zuclopenthixol	Applied when information about dosage and administration route was available
Pimozide	Applied when information about dosage was available
Clozapine	Applied when information about dosage was available
Olanzapine (> 10 mg/d)	Applied when information about dosage was available
Lithium	Applied when information about dosage was available
Risperidone (> 6 weeks)	Applied when information about dosage and treatment duration was available
Aripiprazole	Applied when information about dosage was available
Diazepam	Applied when information about dosage was available
Chlordiazepoxide	Applied when information about dosage was available
Oxazepam (> 60mg/d)	Applied when dosage was available
potassium clorazepate	Applied when dosage was available
Lorazepam (>1 mg/d)	Applied when dosage was available
Bromazepam	Applied when dosage was available
Clobazam	Applied when dosage was available
Prazepam	Applied when dosage was available
Alprazolam	Applied when dosage was available
Ethyl loflazepate	Applied
Cloxazolam	Applied
Hydroxyzine	Applied when dosage was available
Flurazepam	Applied when dosage was available

Estazolam	
Triazolam	Applied when dosage was available
Temazepam	Applied when dosage was available
Midazolam	Applied when dosage was available
Brotizolam (> 0,125 mg/d)	Applied when dosage was available
Loprazolam (> 0,5 mg/d)	Applied when dosage was available
Zolpidem (> 5mg/d)	Applied when dosage was available
Imipramine	Applied when dosage was available
Clomipramine	Applied when dosage was available
Trimipramine	Applied when dosage was available
Amitriptyline	Applied when dosage was available
Nortriptyline	Applied when dosage was available
Dosulepin	Applied when dosage was available
Maprotiline	Applied
Fluoxetine	Applied when dosage and/or treatment moment during the day was available
Paroxetine	Applied
Fluvoxamine	Applied
Bupropion	Applied
Venlafaxine	Applied when dosage was available
Reboxetine	Applied
Methylphenidate	Applied
Pyritinol	Applied
Piracetam	Applied
Ginkgo biloba	Applied
Methadone	Applied
Teofiline	Applied
Codeine (> 2 weeks)	Applied
Dextromethorphan	Applied
Diphenhydramine	Applied
Clemastine	Applied
Doxylamine	Applied
Dexchlorpheniramine	Applied when dosage was available
Dimetindene	Applied
Promethazine	Applied
Mequitazine	Applied
Pseudoephedrine + Triprolidine	Applied
Ebastin	Applied

Table S3. Total Number of PIM Identified In The ADR Reports Received from patients 65 and more years old during 2019.

ATC code	Criteria	DCI	N PIM	%
A02AB04	EU (7)	Dihydroxialumini sodium carbonate	1	0,3%
A02BA02	EU (7)	Ranitidine	2	0,6%
A02BC01	EU (7) / Beers	omeprazole	3	0,9%
A02BC02	EU (7) / Beers	pantoprazole	2	0,6%
A03AA04	EU (7)	mebeverine	1	0,3%
A03FA01	EU (7)/Beers	Metoclopramide	3	0,9%
A03FA03	EU (7)	Domperidone	2	0,6%
A07DA03	EU (7)	Loperamide (> 2 days)	1	0,3%
A07XA04	EU (7)	racecadotril	1	0,3%
A10BB01	Beers	glibenclamide	1	0,3%
A10BF01	EU (7)	acarbose	1	0,3%
A10BG03	EU (7)	pioglitazone	1	0,3%
A10BH01	EU (7)	Sitagliptin	2	0,6%
A10BH02	EU (7)	vildapgliptine	6	1,8%
B01AA07	EU (7)	acenocumarol	7	2,1%
B01AE07	EU (7)	Dabigatran etexilate	24	7,1%
B01AF01	EU (7)	Rivaroxaban	21	6,2%
B01AF02	EU (7)	apixaban	11	3,3%
B03AA	EU (7)	Iron bivalent, oral preparations	1	0,3%
C01AA05	EU (7)/Beers	Digoxin	4	1,2%
C01BC03	EU (7)	Propafenone	1	0,3%
C01BD01	EU (7)/Beers	Amiodarone	34	10,1%
C01EB15	EU (7)	Trimetazidine	2	0,6%
C01EB17	EU (7)	Ivabradine	1	0,3%
C02AC06	EU (7)	Rilmenidine	1	0,3%
C03DA01	EU (7)	Spironolactone (> 25 mg/day)	6	1,8%
C04AD03	EU (7)	Pentoxifylline	1	0,3%
C04AX21	EU (7)	naftidrofuryl	1	0,3%
C05CA05	EU (7)	hidrosmin	1	0,3%
C05CA51	EU (7)	rutoside, combinations	1	0,3%
C07AA05	EU (7)	Propranolol	1	0,3%
C08CA05	EU (7)	Nifedipine	3	0,9%
C08DA01	EU (7)	Verapamil	1	0,3%
G03C	EU (7)	Estrogens	1	0,3%
G04BD02	EU (7)	Flavoxate	2	0,6%
G04BD04	EU (7)	oxybutynin	2	0,6%
H01BA02	Beers	desmopressin	1	0,3%
J01XE01	Beers	Nitrofurantoin	4	1,2%
L02AB01	Beers	megestrol	2	0,6%
M01AB01	EU (7)	indometacin	2	0,6%
M01AB05	EU (7) / Beers	Diclofenac	19	5,6%
M01AB08	EU (7)	etodolac	5	1,5%
M01AB11	EU (7)	acemetacin	1	0,3%

M01AB15	EU (7)	ketorolac	1	0,3%
M01AC01	EU (7)	piroxicam	2	0,6%
M01AE03	EU (7)	ketoprofen	2	0,6%
M01AH01	EU (7)	celecoxib	2	0,6%
M01AH05	EU (7)	etoricoxib	13	3,9%
M01AX17	EU (7)	Nimesulide	1	0,3%
M03BX01	EU (7)	Baclofen	3	0,9%
M03BX08	EU (7)	cyclobenzapine	7	2,1%
M04AC01	EU (7)	Colchicine	2	0,6%
N02AX02	EU (7)	Tramadol	6	1,8%
N03AA02	EU (7) /Beers	Phenobarbital	2	0,6%
N03AE01	EU (7) / Beers	Clonazepam	4	1,2%
N03AF01	EU (7)	Carbamazepine	8	2,4%
N03AX11	EU (7)	Topiramate	2	0,6%
N04AA01	EU (7)	Trihexyphenidyl	1	0,3%
N04AA02	EU (7)	Biperiden	3	0,9%
N04BC05	EU (7)	Pramipexole	2	0,6%
N04BC09	EU (7)	Rotigotine	2	0,6%
N05AA01	EU (7) / Beers	Chlorpromazine	1	0,3%
N05AA06	EU (7)	Cyamemazine	1	0,3%
N05AD01	Beers	Haloperidol	12	3,6%
N05AH02	EU (7)	Clozapine	3	0,9%
N05AH03	Beers	Olanzapine	7	2,1%
N05AN01	EU (7)	Lithium	4	1,2%
N05AX12	EU (7)	Aripriprazole	1	0,3%
N05BA01	EU (7) / Beers	Diazepam	8	2,4%
N05BA06	EU (7) / Beers	Lorazepam	3	0,9%
N05BA08	EU (7) / Beers	Bromazepam	7	2,1%
N05BA12	EU (7) / Beers	Alprazolam	6	1,8%
N05BA22	EU (7)	Cloxazolam	1	0,3%
N05BB01	EU (7) / Beers	Hydroxyzine	3	0,9%
N05CD08	EU (7)	Midazolam	6	1,8%
N05CF02	Beers	Zolpidem	2	0,6%
N06AA04	EU (7)	Clomipramine	1	0,3%
N06AA09	EU (7) / Beers	Amitriptyline	3	0,9%
N06AB03	EU (7)	Fluoxetine	6	1,8%
N06AB05	EU (7) / Beers	Paroxetine	2	0,6%
N06AX12	EU (7)	Bupropion	2	0,6%
N06AX16	EU (7)	Venlafaxine	4	1,2%
R03DA04	EU (7)	Theophyline	1	0,3%
R06AA09	EU (7)	doxylamine	1	0,3%
R06AX22	EU (7)	ebastine	1	0,3%
			N = 337	100,0%

Table S4. Groups / Medicines included in the PIM lists that did not fulfil the criteria due to lack of information to apply it.

Group/Medicines	ATC Identified In Database	N that fulfilled the Criteria	N that were not possible to apply the criteria	% fulfilled
Insulines	94	0	94	0%
Ibuprofen	15	0	15	0%
Naproxen	9	0	9	0%
Risperidone	10	0	10	0%
Proton Pump inhibitors	33	5	28	15%
Lorazepam	6	3	3	50%
Domperidone	2	2	0	100%
Loperamide	1	1	0	100%
Racecadotril	1	1	0	100%
Rivaroxaban	21	21	0	100%
Nitrofurantoin	4	4	0	100%
Etoricoxib	13	13	0	100%
Baclofen	2	2	0	100%
Cyclobenzaprine	7	7	0	100%
Tramadol	6	6	0	100%
Phenobarbital	2	2	0	100%
Clonazepam	4	4	0	100%
Pramipexol	2	2	0	100%
Rotigotin	2	2	0	100%
Haloperidol	12	12	0	100%
Clozapine	3	3	0	100%
Olanzapine	6	6	0	100%
Lithium	4	4	0	100%
Aripiprazole	1	1	0	100%
Diazepam	8	8	0	100%
Bromazepam	7	7	0	100%
Alprazolam	5	5	0	100%
Hydroxyzine	3	3	0	100%
Midazolam	6	6	0	100%
Zolpidem	3	3	0	100%
Nortriptyline	3	3	0	100%
Fluoxetine	6	6	0	100%
Venlafaxine	4	4	0	100%
			Total = 159	
